# A Case of Community-Acquired Elizabethkingia meningoseptica

**DOI:** 10.7759/cureus.45183

**Published:** 2023-09-13

**Authors:** Jae Woo Lee, Bo Sun, Mina Hanna, Ayman Rihawi

**Affiliations:** 1 Medicine, Trinity School of Medicine, Warner Robins, USA; 2 Infectious Diseases, Houston Healthcare, Warner Robins, USA

**Keywords:** community acquired, immunocompromised, bacteremia, cellulitis, elizabethkingia meningoseptica

## Abstract

Many nosocomial infections commonly arise as a result of contaminated water sources in the hospital setting, such as sinks, air-conditioning systems, ventilation devices, and catheters. Among the microorganisms found in these environments is *Elizabethkingia meningoseptica, *a gram-negative bacterium first discovered in 1959 by Elizabeth O. King. This bacterium is a rare cause of meningitis, pneumonia, bacteremia, and skin and soft tissue infections in hospital settings. This case report examines a unique community-acquired transmission of *E. meningoseptica *in a 78-year-old male patient with an extensive medical history who presented with acute fever and confusion coupled with multiple recent falls. Examination and culturing of an open wound on a dry blister of the left lower extremity revealed the presence of *E. meningoseptica.*

## Introduction

*Elizabethkingia meningoseptica* is a gram-negative, non-motile, oxidase-positive, non-spore forming bacterium first discovered in 1959 by Elizabeth O. King, who at the time was studying a novel cause of bacterial meningitis in a pediatric population [1]. *Elizabethkingia supp*. have later been found to possess genes for virulence factors that enable invasion of brain endothelial cells, similar to the factors that contribute to the ability of *Escherichia coli* to cross the blood-brain barrier in neonatal meningitis [2]. Initially, Elizabeth King named it *Flavobacterium meningosepticum*, meaning “the yellow bacillus associated with meningitis and sepsis” in Latin [1]. In 1994, it was reclassified in the genus and named *Chryseobacterium meningosepticum* [3]. However, in 2005, this bacterium was found to be outside the tree of *Chryseobacterium* based on a 16S ribosomal RNA gene sequence study, causing a new genus to be established and named *Elizabethkingia* after the original discoverer [4]. 

According to the Centers for Disease Control and Prevention (CDC), *E. meningoseptica* infections are relatively uncommon, with only five to 10 cases reported each year in the United States [5]. However, there have been an increasing number of reports of complicated infections with this bacterium worldwide. For example, there have been recent cases reported in Qatar, Vietnam, and other countries of *E. meningoseptica* as the causative organism affecting immunocompromised and pediatric patients, leading to prolonged and complicated hospitalizations [6,7,8,9]. With the increased risk of various complications caused by *E. meningoseptica*, heightened clinical awareness is warranted.

*E. meningoseptica *can be found in hospitals' contaminated water sources, such as sink basins, air-conditioning units, mechanical ventilation devices, and catheters [10]. Common symptoms of an infection with *E. meningoseptica* include fever, chills, shortness of breath, cough, and skin and soft tissue infections. Further research has shown that this bacteria can cause bacteremia in immunocompromised patients and premature neonates during hospitalization [11,12]. This report examines the course of hospitalization of a 78-year-old male with a history of type II diabetes mellitus, chronic kidney disease (CKD) stage 3a, and left foot fifth digit amputation secondary to methicillin-resistant *Staphylococcus aureus* (MRSA) infection, who presented with an uncommon community-acquired *E. meningoseptica* infection.

## Case presentation

A 78-year-old male presented to the emergency room with complaints of new-onset confusion and most recent fall on the day of admission. The patient has been weak and shaky to a point of having difficulty holding a cup for a few days with minor falls. The patient related his symptoms to be acute in nature and denied experiencing similar episodes in the past. He noted that recent falls have caused left-sided gluteal pain resulting from falling on his left hip, but he denied any head trauma. As per emergency medical services (EMS), the patient had a fever of 101.0 °F en route to the emergency department. No other complaints were reported by the patient.

The patient has an extensive history of hypertension controlled with losartan 25 mg per os (PO) daily, asthma now resolved, uncontrolled type II diabetes mellitus inadequately controlled with metformin 500 mg PO twice a day (BID), mixed hyperlipidemia controlled with rosuvastatin 10 mg PO daily, CKD stage 3a controlled with torsemide 60 mg PO daily and losartan 25 mg PO daily, gastroesophageal reflux disease (GERD) controlled with famotidine 20 mg PO BID, history of gastrointestinal bleeding resolved, bullous pemphigoid controlled with prednisone 5 mg PO daily and dapsone 100 mg PO BID, and history of anemia requiring blood transfusions. His surgical history includes a left foot fifth digit ulceration that required amputation secondary to an MRSA infection. The patient’s family history was positive for hypertension, diabetes, and arthritis. The patient related a history of anxiety and depression and denied consumption of alcohol, tobacco products, or other recreational substances. Dietary history is unknown and no reported history of any pets at patient's home or in his vicinity. He has no known allergies, and pneumococcal vaccination was given on 2017 and flu vaccine on 2022. 

On examination, the patient was hemodynamically stable, not requiring any supplemental oxygen or pressors. However, he was experiencing mild chills and confusion. Upon arrival at the emergency department, the patient was afebrile with a temperature of 98.1 °F. Physical examination revealed general weakness, difficulty ambulating with slight tremor, and mildly delayed speech, which are not the patient's baseline as per his family. However, the patient was able to answer all questions. The patient was also negative for any dizziness, paresthesia, paralysis, syncope, focal neurological deficits, or pupil abnormality. The patient also denied any gastrointestinal (GI) complaints or change in weight and did not seem malnourished on inspection. Cellulitis of the left lower extremity was discovered. The patient was admitted for in-patient services for further investigation and management. Notably, the patient did have a recurrence of fever of 100.7 °F three days after admission to in-patient services. 

Laboratory testing revealed an elevated procalcitonin level and an increased troponin I level of 0.050 ng/mL (N < 0.030), but serial troponin studies were not conducted in the absence of clinical suspicion of a cardiac episode. Of note, white blood cell count was elevated on admission with an increase in segmented neutrophils and a decreased lymphocyte count, but these values eventually stabilized to normal levels during the course of the patient’s hospital admission and on discharge (Table 1). Red blood cell count, in conjunction with hemoglobin and hematocrit values, were all below normal limits on admission and remained as such during the entire course of his hospitalization (Table 1). Electrolyte abnormalities were noted during the patient’s 18-day hospital stay, but they returned to the patient’s baseline values by discharge (Table 2). Blood urea nitrogen (BUN) and creatinine values were also observed to be elevated on admission, along with an elevated BUN/creatinine ratio (Table 2). Urinalysis was conducted and showed a urine protein level of 100 mg/mmol, along with the presence of blood, leukocyte esterase, and hyaline casts in the urine. Lactic acid was also at 2.2 mmol/L (N: 0.5-1.6).

**Table 1:** Laboratory findings of complete blood counts with differentials conducted daily during the patient’s 18-day course of hospitalization.

	Normal values	On admission	Day 2 post-admission	Day 7 post-admission	Day 14 post-admission	On discharge
White blood cell (x10^3^ uL)	4.5-11	13.6	13.2	5.5	6.6	5.9
Red blood cell (x10^6^ uL)	Male: 4.7-6.1; female: 4.2-5.4	3.43	2.59	2.73	3.08	3.34
Hemoglobin (g/dL)	Male: 13.5-17.5; female: 12.0-16.0	10.0	7.9	8.1	9.0	9.6
Hematocrit (%)	Male: 41-53; female: 36-46	29.2	22.8	22.7	25.9	27.4
Mean corpuscular volume (fm)	80-100	85.1	88.0	83.2	84.1	82.0
Platelet count (mcL)	150-400	142	130	318	434	353
Neutrophils (%)	40-60	-	-	56.6	63.8	54.0
Lymphocytes (%)	20-40	4.0	2.0	23.8	20.6	27.4
Monocytes (%)	4-8	3.0	8.0	7.5	9.8	11.8
Eosinophils (%)	1-3	-	-	9.9	4.1	5.0
Basophils (%)	0-1	-	-	0.7	1.2	1.5
Segmented neutrophils (%)	40-60	89.0	68.0	-	-	-
Band neutrophils (%)	0-5	4.0	21.0	-	-	-

**Table 2:** Laboratory findings of the complete metabolic profile and chemistry panel obtained daily during the patient’s 18-day hospitalization course. BUN, blood urea nitrogen; BUN/Cr, blood urea nitrogen/creatinine; AST, aspartate aminotransferase; ALT, aminotransferase

Day of admission	Normal values	On admission	Day 7 post-admission	Day 14 post-admission	On discharge
Sodium (mmol/L)	135-145	143	141	138	136
Potassium (mmol/L)	3.5-5.1	3.5	4.1	4.0	3.8
Chloride (mmol/L)	96-106	97	112	101	99
Carbon dioxide (mmol/L)	23-29	32	24	33	29
BUN (mg/dL)	6-20	52	15	31	59
Creatinine (mg/dL)	0.6-1.3	1.74	1.07	1.40	1.87
BUN/Cr ratio	10-20	29.9	14.0	22.1	31.6
Glucose (mmol/L)	70-99	117	98	104	99
Calcium (mmol/L)	8.6-10.3	9.6	8.2	9.2	9.2
Phosphorous (mmol/L)	2.7-4.6	-	2.5	-	-
Magnesium (mmol/L)	1.8-2.6	-	2.0	2.0	2.1
Total bilirubin (mg/dL)	Up to 1.2	0.9	-	0.3	0.2
AST (IU/L)	8-33	64	-	15	14
ALT (IU/L)	4.36	20	-	11	10
Alkaline phosphatase (U/L)	20-130	60	-	68	66
C-reactive protein (mg/L)	Lower than 10	35.39	-	-	-
Total protein (g/dL)	6.0-8.3	8.0	-	7.1	7.4
Albumin (g/dL)	3.4-5.4	4.1	2.8	3.6	3.5
Procalcitonin (ng/mL)	Less than 0.05	25.14	-	-	-

**Figure 1:**
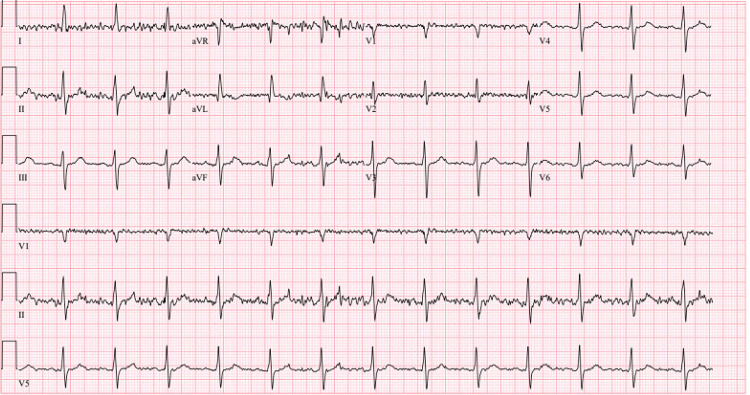
ECG result at the emergency department.

**Figure 2:**
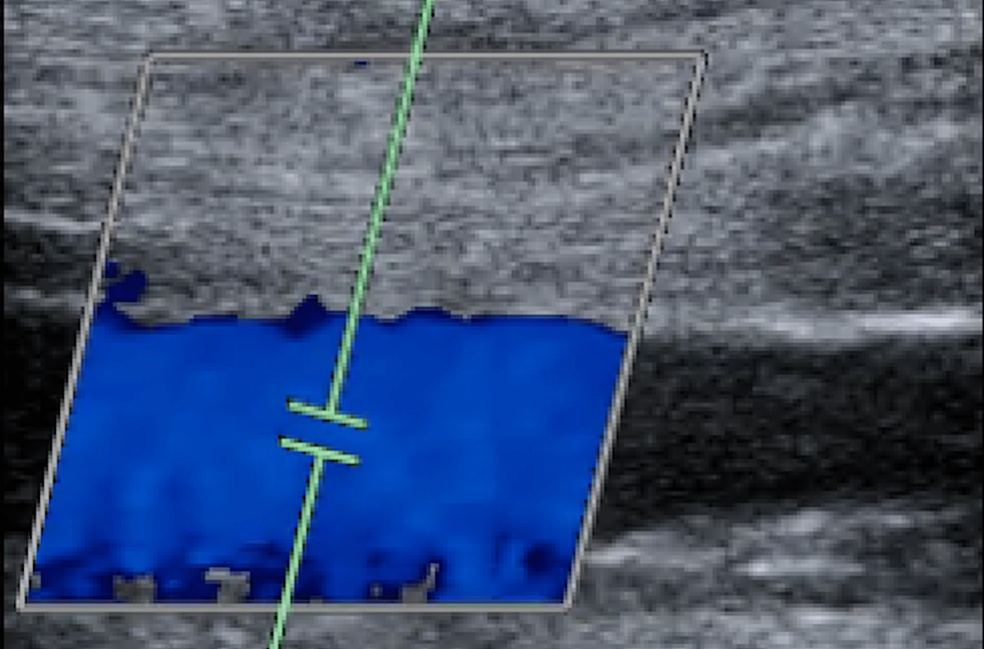
Venous doppler of the left lower extremity.

**Figure 3:**
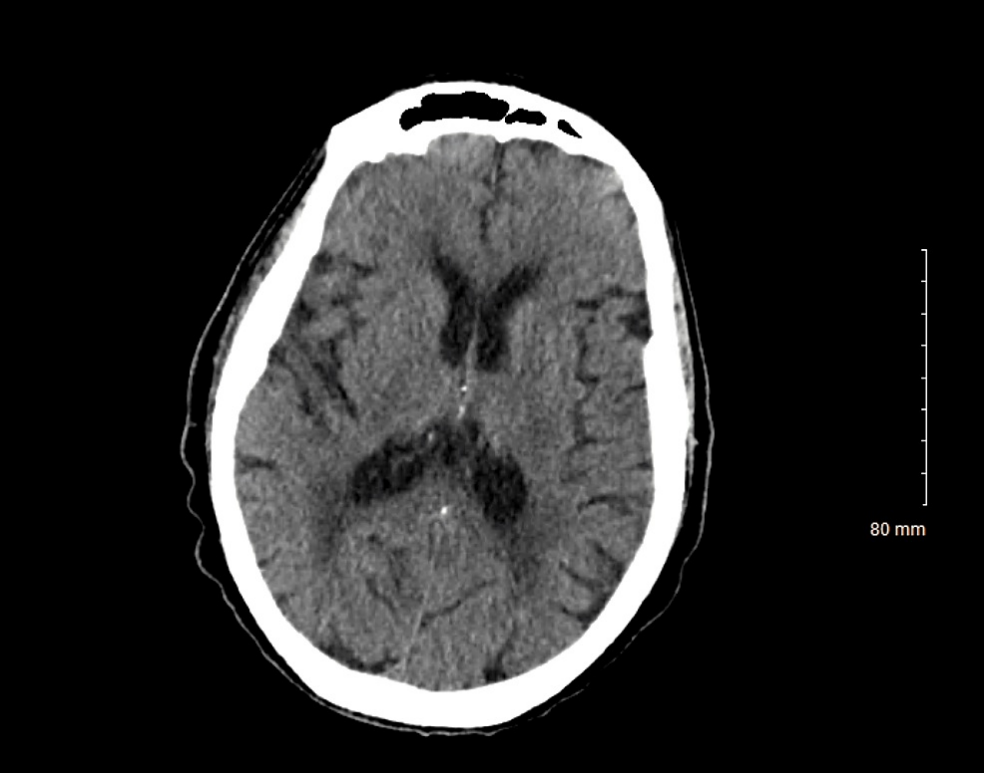
CT scan of the head without contrast.

**Figure 4:**
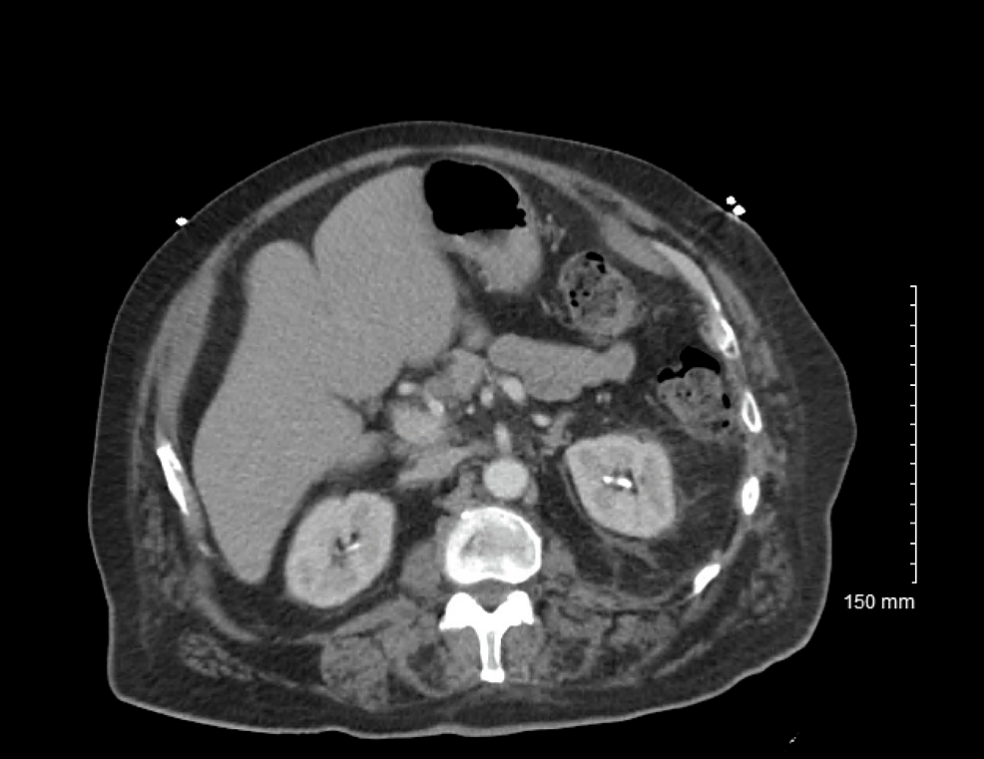
CT scan of the abdomen and pelvis with contrast.

**Figure 5:**
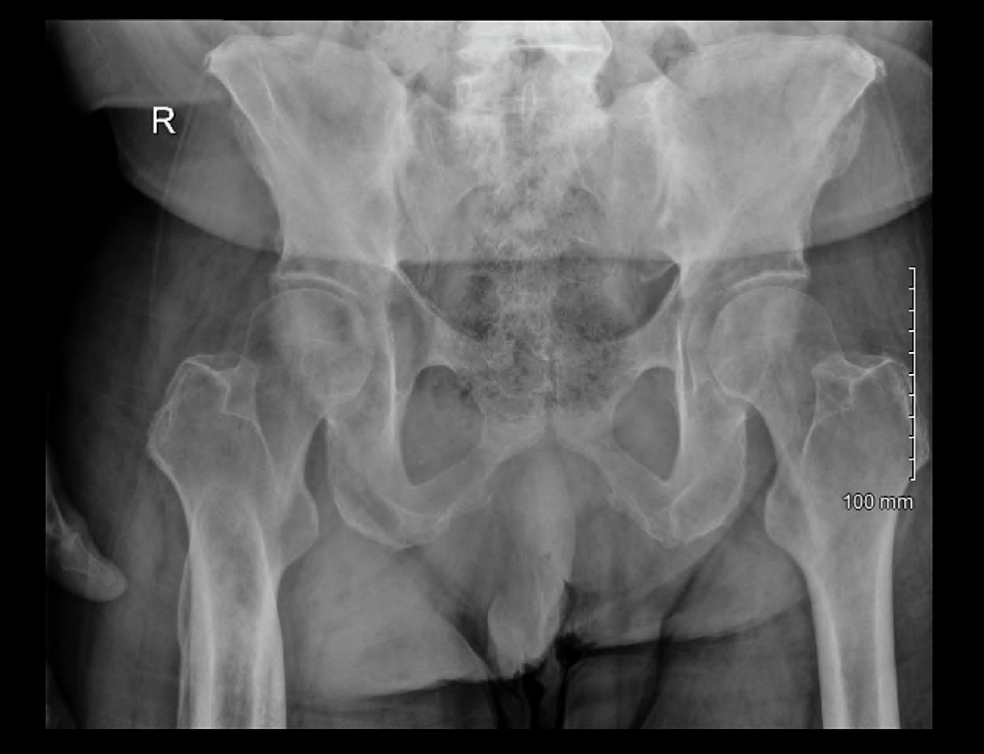
X-ray of the pelvis.

**Figure 6:**
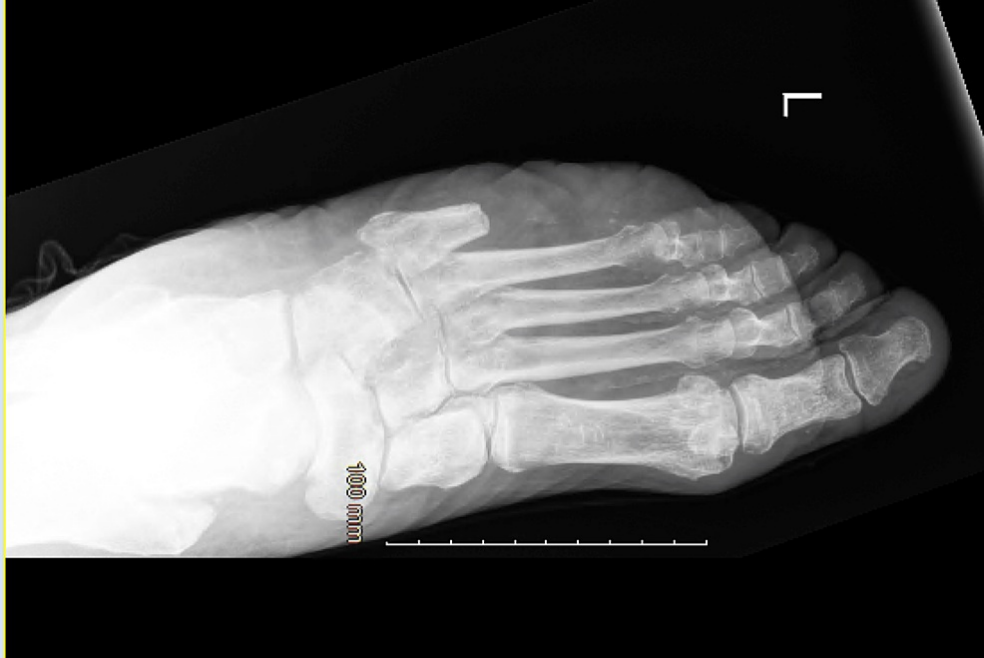
Anteroposterior (AP) view X-ray of the left foot showing previous fifth digit metatarsal amputation.

**Figure 7:**
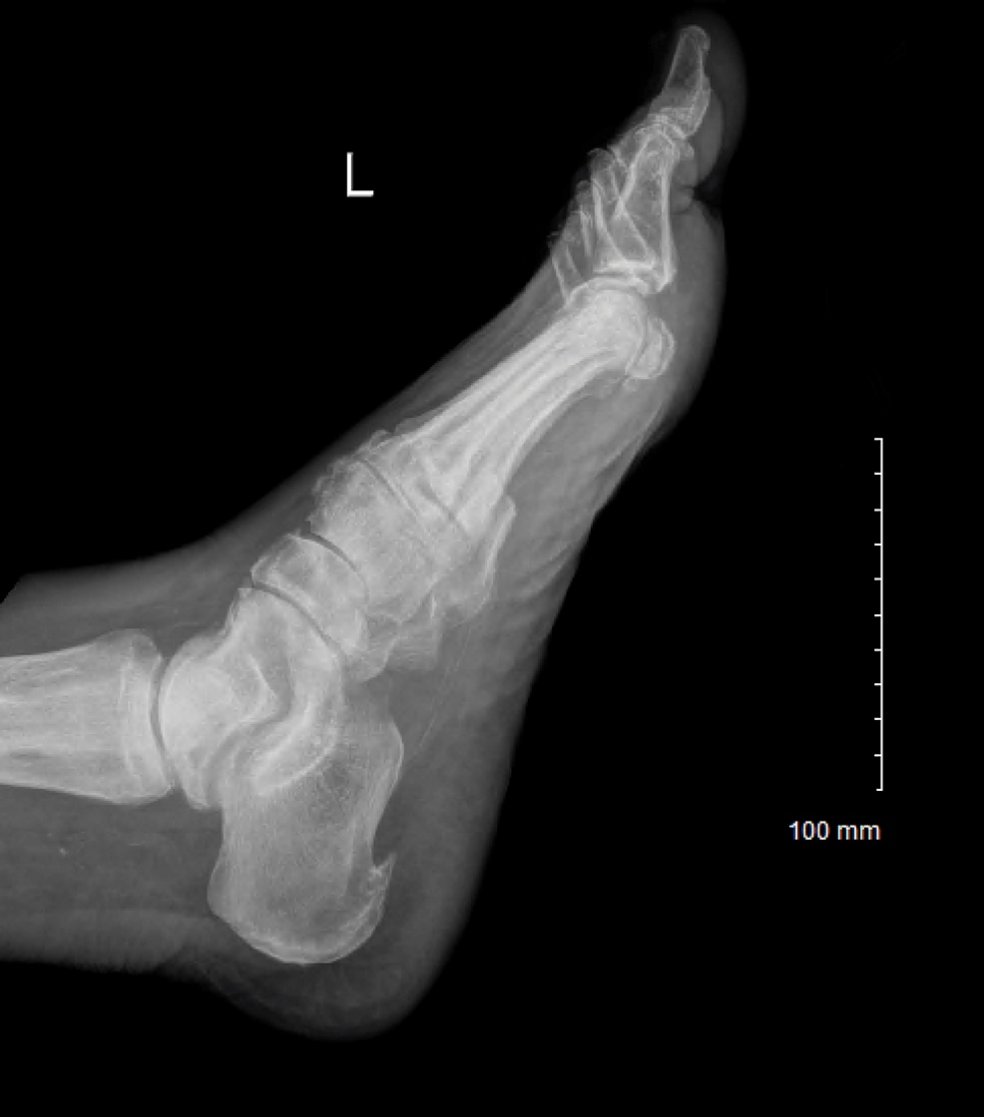
Lateral view X-ray of the left foot.

**Figure 8:**
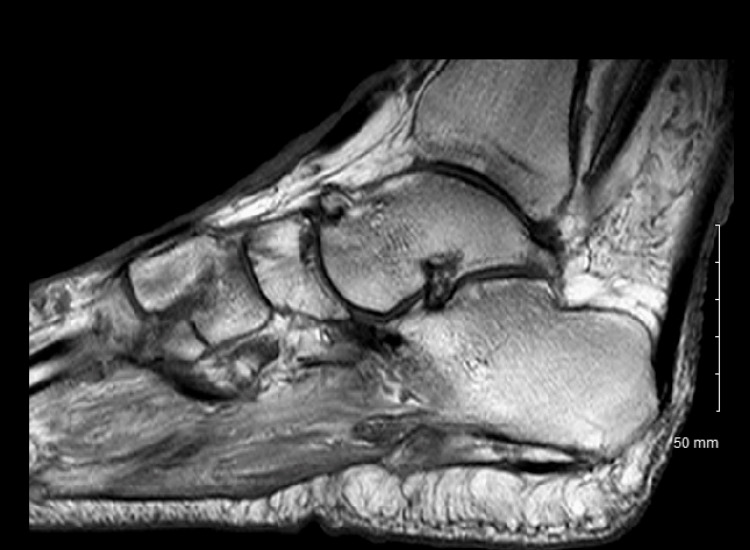
Lateral view MRI of the left foot.

Electrocardiogram (EKG) results were normal, as shown in Figure 1, along with unremarkable findings following venous doppler studies shown in Figure 2. Computed Tomography (CT) imaging of the head (Figure 3), abdomen, and pelvis (Figure 4) were performed to rule out other causes of the patient’s presenting symptoms, and the results were unremarkable. An X-ray of the pelvis (Figure 5) and foot (Figures 6, 7) was also performed, and the results were normal. Lastly, MRI of the left foot was done to rule out osteomyelitis, as shown in Figure 8. No other significant findings were reported. 

The patient met the criteria for sepsis with elevated WBCs, fever, and left lower extremity wound as potential sources of infection. He was immediately started on empiric IV antibiotic treatment with vancomycin and piperacillin/tazobactam therapy. The altered mental status was determined to likely be a result of infectious encephalopathy. Blood cultures were collected, and gram stains revealed the presence of gram-negative bacilli. Infectious Disease was consulted and found multiple small open wounds on the left lower extremity and identified these as a possible source of bacterial entry. Blood culture results showed the growth of *E. meningoseptica *in aerobic cultures. Susceptibility results showed bacterial sensitivity to ciprofloxacin, levofloxacin, and trimethoprim/sulfamethoxazole (TMP-SMX) and resistance to ceftazidime, tobramycin, and gentamicin. The patient was started on levofloxacin therapy and reported decrease in WBCs, absence of fever, negative blood cultures, and clinical improvement of cellulitis.

## Discussion

*Elizabethkingia meningoseptica *bacterial properties 

*E. meningoseptica*, discovered in 1959 by Elizabeth O. King, belongs to the genus *Elizabethkingia*. This bacterial genus typically infects patients who are immunocompromised, such as the elderly and neonatal patients. However, according to a recent study in 2017 by Govindaswamy et al., *E. meningoseptica* can be found in immunocompetent patients as well, with blood cultures of such patients having a marked growth [13]. Notably, the main difference between an infection of *E. meningoseptica* in immunocompromised patients compared to immunocompetent patients is the lack of documented bacteremia symptoms. With this bacteria’s diverse and dynamic infectious profile, it has harbored an increased concern in environments with immunocompromised patients, such as in hospital settings.

This bacterium is a rare gram-negative aerobic opportunistic bacteria that colonizes in water supplies, such as sink basins and water taps [10]. In a recent report done by Chen et al., *E. meningoseptica* has been shown to form biofilms, in contrast to the other species within that genus that do not possess this virulence factor [14]. Biofilms are formed when microorganisms, such as this bacterium, adhere to certain surfaces, often in wet or humid conditions, and secrete extracellular polymeric substances (EPS); these substances, in combination with an exponential reproduction of microbial cells, ultimately form the biofilm [15]. It is, therefore, within reason that *E. meningoseptica* can be found attached to contaminated medical devices, such as ventilators, humidifiers, and intravascular devices [10]. As a result of these characteristics, *E. meningoseptica* infections are more commonly seen as nosocomial infections over community acquired, thus contributing to the rarity and peculiarity seen in this particular case.

Antibiotic profile

*Elizabethkingia spp.* have been shown to possess intrinsic resistance to aminoglycosides, beta-lactam agents, carbapenem, and chloramphenicol. More commonly, these bacteria are sensitive to rifampicin, ciprofloxacin, vancomycin, and trimethoprim-sulfamethoxazole [16]. In a study done by Huang et al., they surmised that the efficacy of fluoroquinolones, when compared with hydrophilic antimicrobial agents, such as beta-lactams, lies in their lipophilic properties. Because of this, fluoroquinolones are able to pass through the blood-brain barrier to reach the bacteria at target locations in patients with meningitis, sepsis, and/or encephalopathies [17]. Knowing this, the decision to initiate levofloxacin therapy proved efficacious, in addition to the given bacterial culture sensitivity and resistance results. However, had this particular case not responded to levofloxacin therapy, the addition of other antibiotic treatments to this current regiment would have been attempted, choosing from the list of agents on the antibiotics sensitivity profile.

Potential explanations and further investigations

As stated above, *E. menigoseptica *primarily infects patients who are immunocompromised, such as this patient with uncontrolled type II diabetes mellitus and long-term steroid use for bullous pemphigoid. In addition, the bacteria spread via contaminated water sources or the formation of biofilms on urinary catheters or ventilation devices during hospitalization. Therefore, our patient’s acquisition of *E. menigoseptica* prior to hospitalization, as the result of blood culture obtained on the day of admission shows, along with his presentation with both cellulitis and bacteremia, further adds to the credibility that this case presentation differs from the common *E. meningoseptica *infectious spread. 

The working hypothesis is that this patient, with his extensive history of uncontrolled type II diabetes mellitus and long-term steroid use, likely acquired *E. meningoseptica* via multiple small open wounds observed in the left lower extremity. This documented cellulitis during the physical examination may have been the entry point by which this patient acquired this bacteria, and his complicated medical history may have contributed to the development of septicemia. The exact source of transmission is unclear, as the pathogen can colonize various water sources in the environment [10]. It is unknown whether there is any pet at the patient's home or in his vicinity. However, there is no reported studies suggesting pets as a source of *E. meningoseptica* infection that warranted further investigation.

Another potential modality of infection, although unlikely, can be attributed to a previous hospitalization two months prior to this encounter, where the patient was admitted for an upper gastrointestinal bleed. This theory would however require that the bacteria remain latent for a prolonged period of time and only became symptomatic at the onset of new wounds, which is highly unlikely. Regardless of how this bacterium was acquired, it is still noteworthy that both cellulitis and bacteremia were simultaneously present in this particular case, which has not been documented before. It should be noted that only few reports on community-acquired *E. meningoseptica* are published, one of which reports of community-acquired pneumonia due to *E. meningoseptica* [18]. This prompts further investigation into the differing methods of transmission of *E. meningoseptica* and the implications it has on public health, both in the hospital setting and in the community.

## Conclusions

This case report presents a 78-year-old male with a right fifth metatarsal amputation from diabetic foot ulcer and long-term steroid use for bullous pemphigoid, presenting with bacteremia and cellulitis from a suspected community-acquired *E. meningoseptica* infection. Due to the low incidence of *E. meningoseptica* infection, this bacterium is not considered among the usual suspects of nosocomial infections. Furthermore, it is seldom a consideration as a culprit of community-acquired infections. Therefore, it is important to highlight the presentation of this bacterium in our patient with the hopes of increasing clinical awareness of *E. meningoseptica* as a rare cause of community-acquired bacteremia and cellulitis in diabetic patients with long-term steroid use.
